# Investigating the Culture Around Sexual Harassment in First-Generation Universities in Southwestern Nigeria: Protocol for a Mixed Methods Study

**DOI:** 10.2196/49126

**Published:** 2023-12-15

**Authors:** Boladale Mapayi, Ibidun Oloniniyi, Olakunle Oginni, Abigail Harrison

**Affiliations:** 1 Department of Mental Health, Faculty of Clinical Sciences College of Health Sciences Obafemi Awolowo University Ile-Ife Nigeria; 2 Brown University School of Public Health Providence, RI United States

**Keywords:** protocol, sexual harassment, SH, universities, support, institutions, Nigeria

## Abstract

**Background:**

The phenomenon of sexual harassment (SH) is a complex issue with multiple prongs that concerns all members of academia and raises serious challenges, particularly regarding prevention and response. SH in tertiary institutions remains a huge problem worldwide, leading to severe emotional, academic, and career difficulties, as well as undue suffering. Institutions have responded in various ways to alleviate the burden of SH with little success, especially in Nigeria. The prevalence is high but reportage is low because of the culture of silence around SH in most educational institutions. This study aims to identify factors associated with SH in tertiary institutions in Nigeria and explore factors surrounding reportage or nonreportage following the experience of SH, the institutional mechanisms to prevent and respond to SH, and the lived experience of survivors of SH.

**Objective:**

The objective of this study was to present a study protocol that is designed to identify factors associated with the experience of SH in tertiary institutions in Nigeria, the institutional mechanisms to prevent and respond to SH, and the lived experience of survivors of SH.

**Methods:**

A mixed (quantitative and qualitative) methods approach is used consisting of a policy review of existing antisexual harassment policies in the selected universities, a quantitative survey to determine the correlates of SH, focus group discussions to explore the perspectives of the university community concerning SH, in-depth interviews to explore the lived experiences of survivors of SH, and key informant interviews to understand the perspectives of people who provide interventions to survivors.

**Results:**

This study was funded in July 2022 by the Consortium for Advanced Research Training in Africa, and data collection started in November 2022. The SH policies were comprehensive, with clear policy statements and definitions, and recognized a wide range of survivors and perpetrators. However, there was no clear mention of prevention and response to same-sex SH. Lived experiences showed negative psychological and social sequelae and little institutional support.

**Conclusions:**

This is the first study that has a component investigating same-sex SH in tertiary institutions in Nigeria. This is also one of the first studies to explore the lived experiences of survivors of SH in Nigerian universities. The findings from this study suggest that periodic evaluation of SH policy implementation will improve institutional support, thus creating safe spaces for survivors and will thereby encourage reportage and support; prevention and response strategies need to be more inclusive; and more interventions should focus on strengthening prosocial skills and healthy, equitable relationships.

**International Registered Report Identifier (IRRID):**

DERR1-10.2196/49126

## Introduction

### Background

Sexual harassment (SH) in educational institutions remains a pervasive problem, and the institutional climate is still the best predictor of the occurrence of SH [1]. SH is not easy to define, partly because it does not involve a homogenous set of behaviors [2]. However, it includes requests for sexual favors and verbal or physical sexual activities in exchange for using or awarding academic marks [3]. When the advances are rejected, a hostile learning environment is created.

There are 2 main types of SH that are recognized in the literature. These include quid pro quo harassment, which is typically exchanging sex for benefits (usually performance or academic favors) or to avoid some detriment (demotion at work or academic failure), and a hostile environment (when speech or conduct creates an intimidating or humiliating environment that negatively affects an individual’s academic or job performance) [4]. Till [5] categorized SH as gender harassment when general sexist or offensive remarks and jokes were made, seductive behavior when inappropriate flirting and sexual advances were made without threat of sanctions, sexual bribery when there was a reward for sexual activity, sexual coercion when the conditions of education were contingent upon sexual cooperation, and sexual imposition also known as sexual assault. Although it is usually easy to identify less ambiguous forms of SH (sexual bribery, sexual coercion, and sexual imposition), it is more difficult to define the more ambiguous forms of SH (gender harassment and seductive behavior), especially gender harassment, which is by far the most prevalent type of SH [1].

The global prevalence of SH in tertiary institutions is high. More than a third of university women in the United Kingdom reported having experienced unwelcome sexual advances (touching, including inappropriate groping) [6]. Nearly 18% of 221 Lebanese women in educational institutions reported being harassed by their professors [7]. In Canada, >80% of the female students surveyed had been sexually harassed in a school setting [8].

In Nigeria, SH came into the limelight through the Cookey Commission panel that was established to address allegations of female students failing examinations for reasons not based on their scholastic abilities [9]. The prevalence of SH in tertiary institutions is higher in Nigeria, ranging from 68% to 80% among female participants [10-12]. The wide range of prevalence rates may reflect the use of diverse instruments to measure SH.

Survivors and perpetrators of SH can be lecturers, students, administrative staff, and any gender [13], although more women than men experience SH and more men than women are likely to be perpetrators [1,8,14]. There are also cases of same-sex SH in tertiary institutions [15], although this experience is less explored. It must be noted that more studies have focused on female students as survivors and men as perpetrators [16,17].

Macrolevel factors for SH range from weak educational systems; low levels of institutional accountability; high population levels of poverty; gender inequality; and poorly trained, underpaid, and understaffed educators [18]. Factors that have been linked to SH in Nigerian universities include contextual societal factors such as gender stereotypes, oppression of women, the patriarchal construct of manhood, the patriarchal configuration of many African societies, communal pressure to secrecy, and the lack of specific policies that address the problem of SH [11,19].

Indecent dressing has been cited in the Nigerian literature as a factor in whether an individual will experience SH or not. This often leads to “victim-blaming.” A common recommendation in the literature is the propriety of dressing for females as a means to prevent SH [12,20-25]. However, confounding factors such as a high sex drive of individual perpetrators have not been fully investigated in this setting [26].

SH in tertiary institutions is a huge concern because of its associated adverse effects [1] such as suboptimal academic fulfillment and withdrawal from academic activities [27,28]; psychological distress with symptoms of depression, stress and anxiety, self-blame, lowered self-esteem, and generally impaired psychological well-being [29-32]; and physical health issues such as headaches, exhaustion, sleep problems, gastric problems, nausea, respiratory complaints, musculoskeletal pain, and weight loss or gain [33,34].

It is known that because of the complexity of SH, the timing, duration, and severity of these effects will vary depending on the individuals affected. Adverse effects are found with gender harassment (the most common) as well as unwanted sexual attention and sexual coercion [35]. These adverse effects seem to be worse when SH is committed by a superior than when committed by peers [36], but it is established that individual reactions vary greatly [37].

SH in colleges and universities is grossly underreported [2]. Survivors rarely report their SH experiences. This is often because of unequal power relations; the fear of loss of status, marks, or job as retaliation; and the associated stigma that it brings [38,39]. Survivors cope with SH in many ways, including by ignoring or appeasing the harasser or seeking social support [1].

The prevalence and associated factors of SH in Nigeria have been investigated [6,7,10,11] but few studies have explored the reasons why SH is underreported and what can be done to improve reporting. In addition, there are no methodical studies on the assertion that indecent dressing is a risk factor for SH [12,20-22], and most studies on SH in Nigeria have focused almost exclusively on heterosexual SH, despite the possible risk of same-sex SH. This study will investigate the culture associated with SH in universities in Nigeria.

This study is based on the integrated conceptualization of SH, founded on the synthesis of the work by Hofstede [40] and Schwartz [41], which explored behaviors directed toward women in different countries and cultures. Luthar and Luthar [42] proposed that the likelihood of experiencing SH perpetrated by men, as well as the tolerance of SH by women, vary between different countries because of the fundamental differences in cultures and values. This study seeks to explore how culture may influence the perception and response to SH experiences in tertiary institutions in Nigeria. However, the study will expand its inquiry to explore how the sociocultural context of same-sex relationships may affect the perception and response of people in same-sex relationships who experience SH in tertiary institutions in Nigeria.

### This Study

This study aimed to identify factors associated with the experience of SH in tertiary institutions in Nigeria, the institutional mechanisms to prevent and respond to SH, and the lived experiences of survivors of SH; explore how the experiences of SH survivors differ between men and women in heterosexual and same-sex relationships; and interrogate how sociocultural contexts and perceptions about dressing, including institutional practices, may influence the experience and responses of survivors to SH perpetrated in tertiary institutions in Nigeria.

## Methods

### Research Design

This study used a concurrent mixed methods design that consisted of a policy review, a quantitative survey, focus group discussions (FGDs), in-depth interviews (IDIs), and key informant interviews (KIIs).

### Study Sites

The study focused on the experiences of staff and students at tertiary educational institutions in Nigeria. There are >170 universities in Nigeria, of which 79 are privately owned and 43 are funded by the federal government [43]. The study recruited participants from 3 public universities owned by the Federal Government of Nigeria, located in southwest Nigeria. The study focused on public tertiary institutions that have existed for >60 years and have a large student and staff population.

The study was also limited to southwest Nigeria because of the homogeneity in the cultural norms and practices of this region. The 3 institutions selected for this study are in the Yoruba-speaking area of southwest Nigeria. Common ties in the language and practices of the community in which the 3 institutions are located may enhance the use of a sociocultural lens to explore the findings of this study.

### Study Population

The target population for this study comprised staff and students of the selected institutions. Study participants were staff or students of the 3 selected universities who were aged >18 years and who gave consent to participate in the study. Staff or students who were severely physically or mentally ill and who lacked the capacity to participate in the study were excluded.

### Quantitative Study

#### Sample Size Determination

The sample size for the survey was computed based on a sample size formula using a margin of error of 2.5%, a confidence level of 95%, and a population estimate of 120,000. The minimum sample per institution was 506 participants and 1518 participants for the study. This was increased to 550 participants per institution to adjust for possible incomplete responses to the questionnaire and to increase the power for subanalyses such as for sex differences. Thus, 1650 participants were surveyed from the 3 institutions using a proportionate sampling technique.

#### Data Collection Procedures

##### Overview

The quantitative survey was developed to explore the perceptions of university community members about SH. The survey focused on the experience of and risk indicators for SH (including mental health determinants) and the reporting of cases within the university community. The survey also explored perceptions of the link between dressing and SH. The questionnaire consisted of questions from validated instruments that measure the phenomena of interest. These were (1) the Sexual Experiences Questionnaire (SEQ) to measure the experience of SH, (2) the Administrator‐Researcher Campus Climate Collaborative tool to measure the campus climate, (3) the Campus Conduct Survey on Sexual Assault and Sexual Misconduct tool to measure the institutional response, (4) pictures and questionnaire items to assess the tendency to perceive a link between provocative dressing and SH, (5) the Changes in Sexual Functioning Questionnaire to measure sexual desire, (6) the Patient Health Questionnaire-4 (PHQ-4) to measure anxiety and depression, and (7) the Suicidal Behavior Questionnaire-Revised (SBQ-R) to measure suicidal behavior.

##### SEQ Measure

The SEQ is the most widely used and validated measure of SH to date [44]. It was developed and validated by Fitzgerald et al [45]. The SEQ has been used in different settings and with different population groups over time. It has been noted that the factor structure of the SEQ (gender harassment, unwanted sexual attention, and sexual coercion) has remained stable across time, culture, and the occupational sector, even when there were variations in the specific items assessing each construct with good psychometric properties [46].

##### Administrator‐Researcher Campus Climate Collaborative Tool

The Administrator‐Researcher Campus Climate Collaborative tool is the result of collaborative efforts by students, legal affairs professionals, campus advocates, campus law enforcement, and sexual assault and harassment researchers [47]. The tool is organized into different modules that allow researchers to determine the length and content of their surveys. They include modules on possible outcomes (which encompasses a standardized 4-item measure of depression and anxiety [PHQ-4]), demographics, campus safety, SH by students and staff (which used questions from the SEQ), and institutional response [46]. All the measures mentioned above were used in this study.

##### Campus Conduct Survey on Sexual Assault and Sexual Misconduct

The Campus Conduct Survey on Sexual Assault and Sexual Misconduct is a survey questionnaire developed by the Association of American Universities [48]. This questionnaire assessed students’ perceptions of experiencing sexual assault or sexual misconduct and their perceptions of institutional response to reporting.

##### The Provocativeness of Dressing

The sexual provocativeness of dressing was assessed by presenting the research participants with 4 pictures, comprising 2 each of a male and a female dressed provocatively and nonprovocatively. Each respondent was shown each of the 4 pictures separately and asked a series of questions concerning them. A total of 2 pictures were shown, each for males and females, respectively. There was 1 picture per sex that depicted a nonprovocative dressing and a sexually provocative dressing. The pictures were taken by a professional photographer using the same background and similar postures as the models. The faces of the models were pixelated to ensure anonymity. The participants were asked to rate the 4 pictures for sexual provocativeness of dressing on a 7-point Likert scale ranging from 1 (not provocative at all) to 7 (extremely provocative) [49,50]. The participants were further asked to indicate the extent to which they expected the individual in the picture to experience SH. This was assessed with the single-item question scored on a Likert scale, such as “How likely is the person in the photograph to be sexually harassed?” (7=likely and 1=not likely).

A pilot study was conducted among 70 students of the Obafemi Awolowo University who were not included in the main study to select the pictures to be used for assessing the sexual provocativeness of dressing. These were selected as follows: 6 pictures each of male and female models in different forms of dressing were taken by a professional photographer with a similar background. The faces of the models were pixelated to ensure anonymity. Each picture was graded based on the level of sexual provocativeness of the dressing on a Likert scale ranging from 1 (not provocative at all) to 7 (extremely provocative). A total of 4 pictures labeled the most provocative and least provocative, 2 each for both males and females were then used in the main study.

Using questions adapted from Moor [51], participants’ perceptions about the motivation of people for wearing provocative clothing as well as their reaction to people wearing such clothing items, were assessed. Participants were instructed to rate motivations for wearing provocative clothes by indicating how much they agreed with 10 statements such as “they wish to feel attractive” or “they intend to convey an interest in sex,” on a 5-point Likert scale from 1 (strongly disagree) to 5 (strongly agree). Participants were also asked about their own customary style of dress and their own motivations for that style. Participants were instructed to respond to 7 questions on a 3-point scale (1 indicating “no,” 2 “sometimes,” and 3 “yes”), such as “Do you dress provocatively in order to feel attractive?” or “Do you dress provocatively to be seductive?”

Furthermore, they were asked about their responses to people who wear what they perceive to be provocative clothing. Items included such questions as “Do you feel that provocatively dressed people are attempting to tempt you?” or “Are you aroused by provocatively dressed people?”

##### Sexual Desire

Sexual desire was assessed using 6 questions from the Changes in Sexual Functioning Questionnaire [52], which assessed sexual desire and ease of arousal. Each item was rated on a 5-point Likert scale ranging from 1 (never) to 5 (always). Higher scores indicated higher sexual motivation and attribution for personal or others’ dressing.

##### PHQ-4 Questionnaire

The PHQ-4 to measure anxiety and depression [53] is a 4-item self-screening measure that combines the items of the Patient Health Questionnaire-2 and Generalized Anxiety Disorder Scale-2, which both contain the 2 core items of depressive and generalized anxiety disorder symptoms. It is used to assess anxiety and depression. It is scored on a 4-point Likert scale, with responses ranging from 0 (“not at all”) to 1 (“several days”), 2 (“more than half of the days”), or 3 (“nearly every day”), with a maximum possible score of 12.

The PHQ-4 compares favorably to the Medical Outcomes Study Short-Form Health Survey subscales in that increasing PHQ-4 scores correlate significantly with declining functional status [53]. It has a high internal consistency, with a Cronbach α of >.80 among the patient population in the United States [53], which compares to a study in Tanzania by Materu et al [54], who reported a Cronbach α of .81. It has good construct validity and has been validated in Germany [55], the United States [56], and Africa [54].

##### SBQ-R Questionnaire

The SBQ-R is a 4-item self-administered questionnaire designed to assess the lifetime and 1-year history, frequency, and severity of suicidal behaviors [57]. Each item in the SBQ-R scale measures a different dimension of suicidal behavior, and it has been found to be straightforward, brief, and easy for participants to complete [58]. The total score on the SBQ-R ranges from 3 to 18, with higher scores reflecting a greater risk of suicidal behavior. The questionnaire exhibited a sensitivity of 80% and a specificity of 91% at a cutoff point of 8 in a sample of adult American psychiatric inpatients [58]. In Nigeria, the questionnaire has been shown to be a valid and reliable suicide risk assessment tool among adolescents and young adults, with a cutoff point of 8 [59].

The data were collected using a secured web-based platform—Google Docs platform. Study participants who were recruited had the option of self-administering the questionnaire using a shared link or using a tablet operated by research assistants (RAs). Students and staff in each institution were categorized by faculties into 3 clusters: Science, Social science, and Arts. In total, 5 Departments were randomly selected from the faculties in each faculty cluster using a ballot. The RAs attended the lecture halls of the selected departments and approached the students. The RAs ensured that all the levels in any chosen department were represented. In addition, links were shared when more than 1 participant was eligible to fill out the questionnaire at the same time. Informed consent was obtained verbally before sending links to the participants, which was also the first question on the web-based questionnaire page.

#### Data Analyses

All quantitative data were coded and entered into SPSS (version 20; IBM Corp) for descriptive analyses. All continuous variables were expressed as means (SD), and categorical variables were summarized using frequencies and percentages. Bivariate and multivariate regression analyses with SH as the outcome variable were used to investigate the relationships between the predictors and SH, as stated in the objectives of the study. Bivariate and multivariate regression analyses with the likelihood of SH as the outcome variable and sociodemographic characteristics, dressing perception, and sex drive as the predictors were performed. For all quantitative analyses, we repeated the analyses with male and female participants to test for sex differences. Differences in sex and sexual orientation were investigated at all levels of the analyses. A *P* value of <.05 was considered statistically significant.

### Qualitative Study

#### Sample Size and Technique

This had two levels:

1. Desk review: a purposive sampling approach was adopted to identify, screen, and select policy documents on SH across the 3 institutions. All the documents were analyzed based on their contents.
2. IDIs, KIIs, and FGDs: participants were recruited for the interviews and the FGDs. Purposive sampling was adopted to identify and recruit key informants from the Directorate of Student Affairs and from members of the antisexual harassment and disciplinary committees in each of the 3 universities. A total of 5 KIIs were conducted in each institution. Both students and staff who had survived SH were targeted for the IDIs. Recruitment was conducted through interactions with the core units of each university, such as the Directorate of Student Affairs, the security unit, the personnel unit, and nongovernmental organizations that have had interactions with SH survivors across the 3 universities. The recruitment and interview processes ensured privacy, confidentiality, and the freedom to share experiences without reservation. In total, 4 IDIs were conducted in each institution. The medium for conducting the interviews (face-to-face, through telephone, or through video calls) was chosen by the study participants.

The FGD participants were randomly selected through multiple calls on identified portals for students and staff at the targeted universities to join a FGD on SH. Participants were chosen based on their consent to participate. Equal gender representation was prioritized. The sample size for the FGDs ranged from 6 to 10 participants per session. A total of 2 student and 1 staff FGD session were held per university.

#### Data Collection Procedures

IDIs, KIIs, and FGDs: The IDIs focused on the experience of SH from the perspective of the survivor, the consequences, whether it was reported, the outcomes, and how the survivor felt about the outcome. The KIIs focused on the perceptions of counselors, SH committee members, and student and faculty on disciplinary committees on SH, institutional response, their experiences, and issues around vicarious trauma. The FGDs were conducted to explore the university community’s perception of SH, its determinants, and how it can be prevented. The focus was also on how dressing is related to SH and the perceptions around same-sex SH.

#### Data Analysis

Desk review consisted of a content analysis of existing SH policies in the 3 selected universities, conducted between September 2022 and February 2023. Common themes and areas of divergence will be highlighted. In addition, the analysis attempted to determine how well the policies aligned with global best practices in the SH field. This was performed by the principal investigator (PI) and coinvestigators.

Analysis of the IDIs, the KIIs, and the FGDs commenced with verbatim transcription of the audio recordings to a Word (Microsoft Corporation) document to avoid misrepresentation and minimize bias. The accuracy, integrity, and completeness of all transcriptions were verified by the PI and coinvestigators by passing them through a 2-level proofreading. At each level, the recordings were listened to and read along with the typed transcripts, ensuring that all discrepancies were corrected. Then, a thematic analysis was conducted, all transcripts were coded by 3 experts, areas of divergence were resolved, and the analysis was managed using NVivo 12 Pro (QSR International). The interviews and the FGDs were synthesized and presented under subheadings to answer the research questions.

### Data Management and Storage

The data collected using web-based surveys was stored on a secure, password-protected laptop. The audio recordings were destroyed immediately after transcription, and deidentified transcripts were saved on a password-protected computer that only the PI, coinvestigator, and statistician had access to. An external hard drive was used as the backup and was stored using a password. The transcripts were shared only with the other members of the research team.

### Ethical Considerations

The research protocol was submitted for ethical review, and approval was received from the Institute of Public Health Research Ethics Committee (IPH/OAU/12/2028), Obafemi Awolowo University, the University of Ibadan Health Research Ethics Committee (UI/EC/22/0313), and the University of Lagos Health Research Ethics Committee (CMUL/HREC/08/22/1082). Written consent was obtained from the participants. Ethics approval at all 3 institutions was obtained between August and September 2022. Preliminary meetings with stakeholders across the 3 universities took place between September and October 2022. Training for the RAs took place in September 2022. Confidentiality was ensured and psychological first aid was offered to interview participants. Furthermore, referrals for therapy were offered to participants who exhibited or reported distress, although none of them took up the offer.

### Knowledge Dissemination

The findings of the project will be summarized and disseminated through the development of institution-specific policy briefs detailing SH issues revealed during the study, which will be given to each institution during a dissemination meeting with recommendations on how to improve prevention and response to SH. In addition, findings from the study will be shared with the scientific community through the presentation of papers at regional or international conference(s) and the publication of scientific papers in peer-reviewed journals. The findings will also be shared with the general public on the project website.

## Results

This study was funded in July 2022 by the Consortium for Advanced Research Training in Africa, and data collection started in November 2022. Ethical approval at all 3 institutions was obtained between August and September 2022. Preliminary meetings with stakeholders across the 3 universities took place between September and October 2022. Training for the RAs took place in September 2022. Content analysis of the antisexual harassment policies of the 3 selected universities took place between September 2022 and February 2023. Recruitment and data collection for both the quantitative survey and the qualitative study took place concurrently between November 2022 and February 2023.

The data from the IDIs has been developed into a manuscript titled “Stifled screams: experiences of survivors of SH in first-generation southwestern Nigerian universities,” which has been published in the *Social Sciences* (Multidisciplinary Digital Publishing Institute).

The SH policies were comprehensive with clear policy statements and definitions, and recognized a wide range of survivors and perpetrators. However, there was no clear mention of the prevention and response to same-sex SH. The forms of SH experienced ranged from physical touch to assault, attempted rape, and rape. The locations of the incidents included the perpetrator’s abode, office, lecture theater, library, and a party. Survivors used several coping mechanisms, including talking with peers, joining social clubs, and heavy drinking. Consequences included not trusting others, blaming themselves, and feeling sad, suicidal, angry, and guilty.

The data analysis for the quantitative survey is ongoing. Dissemination meetings for the selected universities, presentations at scientific conferences, and subsequent publications in peer-reviewed journals are expected to be completed before the end of 2023.

## Discussion

### Principal Findings

To our knowledge, this is the first study that has a component investigating same-sex SH in tertiary institutions in Nigeria. It is also one of the first studies to explore the lived experiences of survivors of SH in Nigerian universities. This study hypothesizes that there will be a high prevalence of SH in the selected institutions; however, there will be underreportage of SH, and many students will find the institutional measures inadequate to curb SH. Globally, most studies have shown mixed results on the prevalence of SH in tertiary institutions [10,60-65]. Numerous studies have shown that both males and females are survivors of SH, although females are more affected [66,67]. Factors that influence nonreportage among students include shame, fear of retribution by the lecturer, and godfatherism, among others [38,39,68]. There has been extensive research regarding dressing and SH [12,20-25,51], with mixed results; some studies found that provocative dressing increased the likelihood of an individual being sexually harassed [69,70].

Although the 3 universities have antisexual harassment policies, reports of SH still appear in the news from these universities [60-62] signifying that the existence of SH policies is not enough to curb SH. It is hoped that the dissemination of content analysis will emphasize the need to supervise the implementation of institutional SH policies to improve prevention and response. In addition, the reports from lived experiences highlighted survivors’ experiences of SH, the mental health consequences, how they have coped, the responses from their institutions, and barriers to reporting both to the institutions and law enforcement agencies. It has been shown that the lived experiences of survivors can be a powerful tool for advocacy and action in the response to SH [66]. The survey focused on the prevalence and correlates of SH, focusing on gender differences and differentiating between same-sex and heterosexual SH. It also examined the campus climate and how it influenced the experience of SH for the campus community.

### Limitations

One of the limitations of this study is that the quantitative aspect is cross-sectional and cannot be used to determine causality. The qualitative aspect of the study regarding the lived experiences of survivors is retrospective in nature and may be subject to recall bias.

### Conclusions

It is hoped that the findings from this study will improve our understanding of the factors that drive SH among staff and students in tertiary education institutions in Nigeria and the risks susceptible populations face. This information will help with the design and implementation of policies and programs that can reduce the risk of susceptible populations to SH, in view of the long-term and or lifetime mental health consequences and social effects of SH on survivors and improve reportage among survivors. Future research needs to focus on contextualized interventions for the prevention and response to SH in tertiary institutions.

## Abbreviations

FGD: focus group discussion
IDI: in-depth interview
KII: key informant interview
PHQ-4: Patient Health Questionnaire-4
PI: principal investigator
RA: research assistant
SBQ-R: Suicidal Behavior Questionnaire-Revised
SEQ: Sexual Experiences Questionnaire
SH: sexual harassment

## Data Availability

The data sets generated during this study are available from the corresponding author upon reasonable request.
